# A Systematic Review of Precision Livestock Farming in the Poultry Sector: Is Technology Focussed on Improving Bird Welfare?

**DOI:** 10.3390/ani9090614

**Published:** 2019-08-27

**Authors:** Elizabeth Rowe, Marian Stamp Dawkins, Sabine G. Gebhardt-Henrich

**Affiliations:** 1 Department of Zoology, University of Oxford, Mansfield Road, Oxford OX1 3SZ, UK; 2 Center for Proper Housing: Poultry and Rabbits, Division of Animal Welfare, University of Bern, Burgerweg 22, CH-3052 Zollikofen, Switzerland

**Keywords:** precision livestock farming, poultry, health and welfare, systematic review

## Abstract

**Simple Summary:**

Precision livestock farming (PLF) is the use of technology to help farmers monitor and manage their animals and their farm. This technology can help to improve animal welfare by enabling farmers to act as soon as any problem arises. However, the technology can also be used to increase production efficiency on the farm, which could be prioritised over the animals’ welfare. The aim of this study was to give an overview of PLF technology development in poultry farming, and to investigate whether improving welfare has been the main goal of PLF development. The results suggest that PLF development in poultry farming so far has focussed on improving animal health and welfare, more so than increasing production. However, despite the interest in PLF research for poultry farming across the world (especially in the USA, China and Belgium), most of the technology is still being developed (prototypes); only a few are available for farmers to buy and use. This means that future work should focus on making these technologies commercially available to farmers, so that systems developed to improve welfare can be used to improve the welfare of farmed birds in the real world.

**Abstract:**

Precision livestock farming (PLF) systems have the potential to improve animal welfare through providing a continuous picture of welfare states in real time and enabling fast interventions that benefit the current flock. However, it remains unclear whether the goal of PLF development has been to improve welfare or increase production efficiency. The aims of this systematic literature review are to provide an overview of the current state of PLF in poultry farming and investigate whether the focus of PLF research has been to improve bird welfare. The study characteristics extracted from 264 peer-reviewed publications and conference proceedings suggest that poultry PLF has received increasing attention on a global scale, but is yet to become a widespread commercial reality. PLF development has most commonly focussed on broiler farming, followed by laying hens, and mainly involves the use of sensors (environmental and wearable) and cameras. More publications had animal health and welfare than production as either one of or the only goal, suggesting that PLF development so far has focussed on improving animal health and welfare. Future work should prioritise improving the rate of commercialisation of PLF systems, so that their potential to improve bird welfare might be realised.

## 1. Introduction

The term ’Precision Livestock Farming’ (PLF) was coined at the start of the 21st century, with the first conference on PLF held in 2003 [1]. Since its inception, many different definitions of the term have been generated and used in the literature. Based on the main shared aspects of the various definitions, the authors generated the following definition: PLF is the use of technology to automatically monitor livestock, their products and the farming environment in real time, in order to aid farm management, through supplying the farmer with relevant information on which to base management decisions, or by activating automated control systems.

The ’precision’ in PLF can be considered to refer to control of processes. Through more precise control over production processes, PLF can help farmers to improve their productivity and profitability. Banhazi and Black [2] argue that a major benefit of adopting a PLF system is to ensure that “every process within a livestock enterprise, which can have a large positive or large negative effect on productivity and profitability, is always controlled and optimised within narrow limits.”

As well as the potential to increase production, PLF technologies have the potential to improve animal welfare. PLF allows for non-intrusive assessment of welfare, where information can be collected without the stress of disturbing or handling animals [3]. Continuous monitoring can also provide a more complete picture of the overall welfare state of animals, rather than a snapshot in time as offered by traditional human assessment [4]. Alerting farmers to problems as they arise in real time allows for fast and targeted interventions which will benefit the current flock, compared to traditional welfare assessments that occur at the end of the production cycle [4]. PLF technology may offer more objective measures of welfare than traditional assessment methods carried out by human observers. Banhazi and colleagues [5] argue that “PLF can greatly contribute to an objective discussion on animal welfare by providing real data to the otherwise very subjective (and sometimes emotional) discussion process.” Finally, Werkheiser [6] puts forward that PLF allows “modern, large-scale farms to replicate and even to improve on the benefits of caring farmers who know their animals, transferred to a much larger scale. This could be done via closer monitoring than farmers can provide to even a few animals, as well as integration of the data via decision algorithms that improve on the guesswork of traditional stockpersons.” There is, however, a concern amongst animal welfare advocates that PLF systems, in aiding the management of intensive farming systems, may entrench the use of such systems that have limited potential for achieving good welfare outcomes, and that PLF will be used as a substitute for addressing the root causes of welfare issues [7]. On the other hand, PLF technologies can serve to highlight the welfare issues of poor systems and inform evidence-based strategies for their improvement. PLF can only be part of a solution to improve welfare, alongside other interventions to address the root causes of issues, for example in broiler farming using slower growing strains, reducing stocking density and increasing the dark period length, which have been identified as factors contributing most to broiler welfare [8].

While the potential of PLF to improve animal welfare has been discussed, what remains unclear is whether improving welfare has been the goal of PLF development in poultry, or if the focus has been on increasing production efficiency. These two factors are not mutually exclusive; improvements in welfare can be linked to improvements in production, for example by reducing mortality [9]; therefore, PLF systems can aim to improve both animal welfare and production. What is important from an animal welfare perspective is that PLF is not intended to increase production at the expense of animal health and well-being.

Poultry farming, and in particular broiler farming, is an important area in which to focus efforts on improving welfare, because of the sheer number of animals involved and the potential for improvements in their welfare. Modern broilers suffer from problems such as sudden death syndrome, ascites, lameness and contact dermatitis as a result of their fast growth rate [10,11], which has increased through breeding programmes by 400% since the 1960s [12]. Broilers are the world’s most numerous bird, with a standing population of 22.7 billion, an order of magnitude greater than the standing stocks of any other farmed species [13]. The highest farm animal numbers are found in poultry operations, with up to tens of thousands of individuals in one barn. Each individual animal is worth relatively little and the turnover of flocks is very fast, with modern broiler strains in conventional intensive production systems reaching their target weight in just 5–6 weeks or less [14]. This means that concern for the welfare of an individual bird may be low. The profit margin for poultry farmers is small, creating further conflict between production and bird welfare.

While the number of chickens farmed for meat is already huge, meat consumption is currently predicted to increase, not only because of a growing human population, but also because of increasing incomes and urbanisation [15]. This means that poultry farming is set to increase in low income countries where animal welfare may not yet be seen as a priority. Poultry meat consumption specifically has grown in comparison to other meat types. In the OECD (Organisation for Economic Co-operation and Development, Paris, France), an organisation of 36 countries, beef pork and sheep meat consumption levels have varied very little between 1990 and 2017. Poultry meat consumption on the other hand has increased by 70.5% [16].

Therefore, poultry welfare is an especially important area to focus efforts on improving welfare, and PLF is one potential tool to achieve this (in addition to improving farming practices in other ways), through enabling continuous monitoring and fast interventions benefiting individuals in their lifetime. The question remains, is PLF currently fulfilling this potential to improve bird welfare, or has increasing production efficiency been the driver behind PLF development in the poultry sector?

The aim of this systematic review is two-fold. Firstly, we aim to present an updated overview of the research conducted on PLF technologies in the poultry sector, to assess how much PLF is currently being used in poultry farming. Secondly, we ask whether the development of PLF for poultry has so far focussed on improving bird welfare, or if increasing production efficiency been the main driving factor.

## 2. Materials and Methods

### 2.1. Definition of Precision Livestock Farming

From the different definitions of PLF that have been used in the literature, the working definition of PLF that was developed and used in this review is: the use of technology to automatically monitor livestock, their products and the farming environment in real time, in order to aid farm management, through supplying the farmer with relevant information on which to base management decisions, or by activating automated control systems.

Therefore, to be considered as a PLF system according to the authors’ working definition, the system had to be automated at least in part, if not fully. For the purpose of this study, livestock and their products refer to living birds and eggs only; monitoring of carcasses, meat or manure were not included. Technology used to monitor birds prior to hatching, e.g., sexing of embryos was also excluded. Publications concerning the development of models or algorithms for use in PLF systems, such as computational fluid dynamics (CFD), machine learning, etc., were not included; although these are aspects of PLF systems, they do not fall under the authors’ working definition of PLF. In addition, publications concerning traceability, for example vehicle identification during the transport of poultry or poultry products, or data management systems for the traceability of information between each link of a poultry production chain, were not included. Although these can be considered as applications of PLF technology, they do not fall under the working definition of PLF used in this review.

### 2.2. Literature Search

A systematic search, following PRISMA guidelines [17], for published peer-reviewed literature and conference proceedings on the use of PLF technologies in poultry farming was carried out between 1 February and 3 April 2019. Searches were performed in the following databases: CAB Direct, PubMed, Scopus and Web of Science.

### 2.3. Selection of Search Terms

We selected 18 terms relating to PLF, and 11 poultry-related terms. Quotation marks were used to narrow the search to the exact phrase when the term contained common words.

Although review papers were excluded from the results (see eligibility screening below), reviews on the use of technology in poultry farming [18,19,20,21] were screened for relevant references. If there were references that had not been returned by the search terms used up to this point, search terms were generated from these papers. This process led to 14 additional terms related to PLF, and searches were conducted using these terms (Table 1).

### 2.4. Search Strategy

The search fields were “topic” (covering title, abstract, author keywords and keywords plus) in Web of Science, “article title, abstract, keywords” in Scopus, and “all fields” in PubMed and CAB Direct.

The poultry terms were combined with parentheses and the Boolean operator OR, and each precision farming term was combined in turn with the poultry terms using AND; for example: precision livestock farming AND (poultry OR chicken OR chick OR laying hen OR broiler OR pullet OR duck OR goose OR turkey OR hatchery OR slaughter).

For several of the precision farming terms, PubMed returned over 100 results; in these instances, the filter “other animal” was used to filter out irrelevant human-related studies. For several search terms, Web of Science also returned more than 100 results; in these instances, relevant categories were chosen from the list of Web of Science to filter for relevant results.

### 2.5. Eligibility Screening

Three inclusion/exclusion criteria were used to screen each search result. The publication had to describe novel research (no reviews) on PLF technologies used in poultry according to the above definition, in English, German, or French.

One author (ER) carried out the literature search and screening process. After the screening process, 20 papers were randomly selected using a random number generator [22], and agreement was checked between all authors on whether these met the inclusion criteria. If there were any papers the first author was unsure about during the screening process, they were discussed with the other authors until a decision was reached.

### 2.6. Study Characteristics

Results were categorised according to the headings in Table 2, using information from the abstracts, and from the full text where available if the abstract was insufficient. Apart from the categories ’prototype or commercially available system’ and ’year’, papers could be classed in more than one category, meaning that percentages did not sum to 100%. Income groupings of countries (a development indicator) were based on the latest World Bank data [23]. Because of the large number of countries of author affiliation, any countries with under five publications were categorised together under ’other’; a full list of countries is given in Appendix A. PLF systems were classed as commercially available if they were available for purchase as a complete system or used commercially available sensors. The heading ’goal’ describes the study’s goal(s): whether this was to improve animal health and welfare, human health, production or sustainability. These categories are not mutually exclusive and studies could have more than one goal; therefore, publications could be classed in more than one category. Where the goal of the study was not stated explicitly, it was inferred from the keywords and the information in the abstract or full text. Where there was insufficient information to make this inference possible, the category was left blank, leading to a small amount of missing data. The category sensor included both sensors for environmental monitoring (e.g., temperature, humidity), as well as wearable sensors [24]. Wearable sensors included Radio-Frequency Identification (RFID) systems, which can be used as movement sensors [20].

For publications which had animal health and welfare as the only goal of the study, the type of parameters measured by the PLF system (i.e., used as welfare measures) were extracted.

## 3. Results

### 3.1. Study Selection

**Figure A1:**
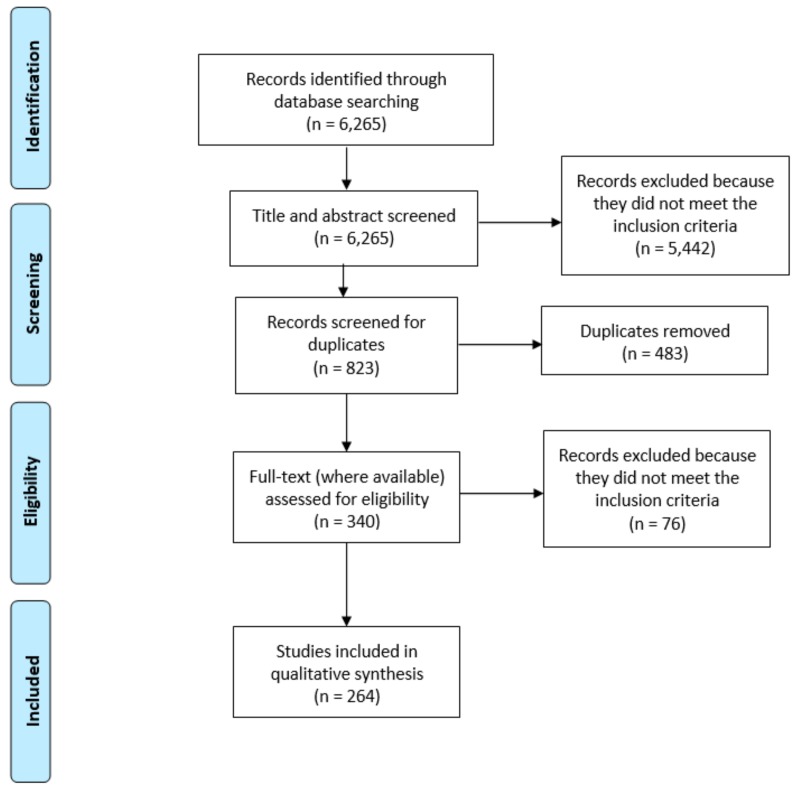
Modified PRISMA flow diagram illustrating the systematic review search strategy and study selection.

A total of 6265 results were returned by the search strategy and screened for eligibility. This resulted in 264 papers that were included in the review, 203 of which the authors had access to the full text (see Figure A1 for PRISMA flow diagram).

### 3.2. Study Characteristics

**Figure 1:**
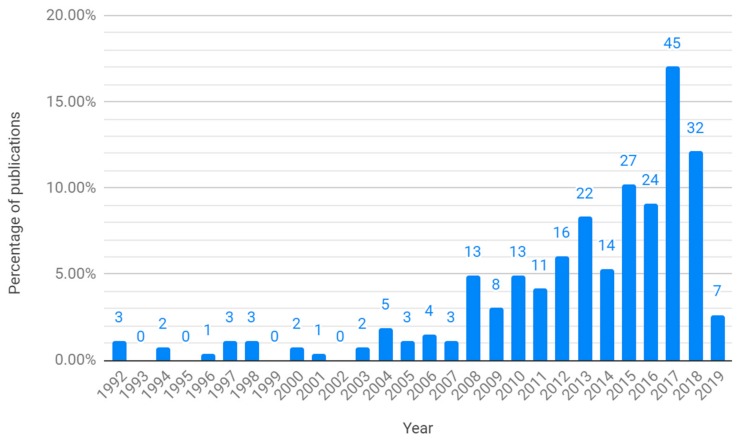
Number (data label) and percentage of publications by year. Publications in 2019 are up until 3 April.

Figure 1 and Table 3, Table 4, Table 5, Table 6, Table 7 and Table 8 describe the study characteristics extracted from the search results. The first papers on technology which can be classed as PLF in poultry farming, according to the authors’ definition, were published in 1992 (Figure 1). The number of publications remained low until a marked increase in 2008, reaching a peak in 2017. Authors of the publications were affiliated with institutions from a total of 40 different countries (Table A1). The biggest proportion of studies was contributed to by at least one author from the USA, followed by Belgium and China (Table 3). The majority of publications were authored by at least one author from a high-income country; there were no publications with authors from a low-income country (Table 4). Half of the studies described sensor technology, and over a third described the use of cameras; microphones represented a smaller proportion (Table 5). The vast majority of studies described prototype systems (96.21%, *n* = 254); only 10 papers described commercially available systems (3.79%). The largest proportion of papers described PLF technology in broiler farming, followed by laying hens (Table 6). The largest proportion of publications had animal health and welfare as one of the goals of the study, followed by production, which was one of the goals of over half the publications (Table 7). A total of 105 papers (39.77% of all publications) had animal health and welfare as the only goal of the study, compared to 72 papers (27.27%) with production as the only goal. For the PLF systems with animal health and welfare as the only primary goal, most of the measurements used to assess animal health and welfare were behaviour-based; the largest proportion of publications used locomotory behaviour as a measure of welfare, followed by vocalisations or bird sounds (Table 8). The category ’acceptability’ in Table 8 does not describe parameters measured directly by a PLF system, but instead concerns the acceptability to farmers of the PLF technology for studies where the goal was to improve animal health and welfare. These studies included: the acceptance of PLF technology by farmers in the EU-PLF project [25], the development of power-saving sensors [26], investigating birds’ reactions to the use of robots [27], the effect of wearable sensors on bird behaviour [28,29,30] physiology [29,30] and health [30], the effect of precision feeding systems on bird behaviour [31,32], and methods to extract chicken images from background noise in image analysis [33].

## 4. Discussion

The first aim of this review was to provide an overview of the current state of PLF research in poultry farming, to assess the progress of this field since its inception. Previous reviews on PLF technologies in poultry farming [18,19,20,21] have given illustrative examples of technological developments in this field, but have not provided a comprehensive overview of all research in this area. By conducting a systematic literature search, the current review provides a more complete picture of the state of PLF development in the poultry sector thus far. Technology is developing rapidly in this field; this review provides an update on new PLF systems published subsequent to the previous reviews. The second aim was to investigate whether the focus of PLF research has been to improve bird welfare, because whilst the potential for PLF to increase welfare through improved monitoring has been discussed, there remains a risk that PLF will be utilised to prioritise production efficiency, which could come at the expense bird welfare.

Based on the results of the systematic literature search, research into PLF technology for poultry farming did not take off until the late 2000s. Since then, research output in this field has tended to increase up until 2017, waning slightly in 2018. This suggests a growing interest and investment in poultry PLF research. Evidencing the continued interest and investment in PLF technologies in the poultry sector, the Foundation for Food and Agriculture Research (FFAR) and McDonald’s Corporation have recently launched ’SMART Broiler’, a research grant of $4 million to drive the development and commercialisation of automated monitoring tools to assess broiler welfare [34].

Poultry PLF research has not been confined to a small number of countries; contributors to the publications were affiliated with institutions from 40 different countries (Table A1). There were no authors affiliated with low-income countries, and the majority of publications were authored by at least one author from a high-income country. However, over a third of publications (37.88%) had at least one author affiliated with an upper-middle-income country, and 4.92% a lower-middle-income country. This suggests that it is not only the wealthiest countries that are interested and investing in PLF for poultry farming.

The main countries producing poultry PLF research were the USA (18.94% of publications had at least one author from the USA), followed by Belgium (18.56%), and China (17.05%). The USA is the world’s largest producer of poultry according to the most recent available data (20 million tonnes produced in 2014), followed by China (18 million tonnes produced in 2014) [35]. Therefore, it is logical that these countries are interested in the development of technology that could improve poultry farming. Belgium is the country where early pioneers of PLF were based [5] and continue to be active, potentially explaining why Belgium is one of the major contributors to poultry PLF research. It should be noted that only papers published in English, French or German were included in the review, as these were the languages that could be understood by at least one of the authors. Excluded papers in different languages, such as Chinese, could have altered the results of this review.

More PLF technology has been developed for broilers (43.18% of publications) than any other bird type, a finding that is in line with the fact that broilers are the most commonly farmed type of poultry [13]. Broilers may also be the bird type of interest in PLF development as the scope for broiler welfare improvement is great (as discussed earlier in this review). As laying hens are also a commercially important type of poultry, it follows that laying hens were the second most common type of bird for which PLF technology has been developed (25.38% of publications). Egg consumption is high in many countries around the world: in 2013, egg consumption per capita was 18.65 kg in China (one of the highest levels in the world), 14.58 kg in the USA, and 12.59 kg in Belgium [35].

The majority of PLF in poultry involved the use of sensors (51.89%), although cameras were used in a large proportion of the studies (42.42%). The use of microphones appears to be less popular in poultry PLF (14.02%). That over half the publications involved the use of sensors may be explained, at least in part, because this was the broadest category of technology. The category sensors included not only sensors to monitor environmental parameters such as temperature and humidity, but also ’wearable sensors’ [24]. This included RFID; although RFID is used for individual identification of animals, this technology can also be used as movement sensors [20], and used track behaviour, including locomotory behaviour. For example, the time difference between an RFID-tagged bird passing two RFID readers and the distance between these readers enables movement speed to be calculated, and behaviours such as time spent feeding and resting can also be monitored (e.g., [36]). As another example, RFID has been used to sense when a hen enters or exits a nest box which, along with a pressure sensor to detect the presence of an egg, has led to the design of a smart nest box to monitor the laying performance and behaviour of hens [37]. Previous reviews have noted a growing interest in wearable sensors for animal health management [24]. Environmental sensors are easier to interpret than cameras and microphones: the output of an environmental sensor such as a thermometer delivers the parameter of interest directly (a temperature reading), whereas the output of a camera or microphone must first be analysed and interpreted before the parameter of interest (for example locomotory behaviour) is produced. This may explain the apparent popularity of sensors (at least environmental sensors) in PLF systems.

Almost all papers (96.21%) described prototype systems, suggesting that there are very few PLF systems for poultry farms that are currently commercially available. The commercially available technologies were: the eYeNamic™ camera system [38,39], and environmental sensors to measure temperature [40,41,42,43], ambient dust [44], relative humidity [41,42,43], vibration [45], ammonia concentration [46], carbon dioxide concentrations [41,46], and a thickness and crack sensor for eggs [47]. The eYeNamic™ camera system is produced commercially by Fancom BV and collects and processes images in order to monitor chickens’ distribution and activity, which “can be conceived as valuable indicators of animal welfare” [38]. It should be noted that some of the prototype systems in this review used the commercially available eYeNamic™ cameras, but as the systems themselves were prototypes the publications were categorised as such (e.g., [48]). Conversely, publications that involved the use of commercial sensors described investigations of where best to place these sensors, and so could be categorised as commercially available systems (e.g., [41]). It should also be noted that some of the prototype systems could have become commercially available since the time the study was published. Nonetheless, this result suggests that the application of PLF technology in poultry farming is still a future prospect rather than a current reality.

The evident interest in the use of PLF for poultry farming raises the question: why are there not more commercial PLF systems in place on poultry farms? Wathes [49] suggests that PLF technologies remain uncommon because research does not involve manufacturing companies from the start. Such companies could help to develop specifications for commercial success. In addition, few systems undergo trials under commercial conditions, and these are vital in order to show technical success to farmers and other stakeholders [49]. Incomplete development of technology, especially when equipment shows poor robustness and reliability, will lead to rejection by early adopters [3]. It is also not clear whether there is a demand for new monitoring technologies from farmers [49], and farmers may lack confidence in technology-based production systems [3]. Furthermore, the payback period for farmers investing capital in PLF systems is uncertain [3].

The obstacles discussed above apply to PLF technologies in general, for all farmed species. However, it appears that PLF in the poultry sector lags behind that of other species, for example dairy cattle. Commercially available PLF technology in the dairy sector includes devices to identify, track and milk individual animals, feed animals automatically, and obtain diagnostic data about a range of health and performance related criteria [50]. The dairy sector has had a longer history of PLF development than the poultry sector: the first widespread application of PLF was the individual electronic milk meter for cows, which became commercially available in the 1970s [51], followed by automated oestrus detection devices in the 1980s [52], both decades before the term PLF was coined.

Caja and colleagues [50] suggest that “dairy farmers will pay for and use technologies that provide what is, to them, a straightforward answer to a straightforward question (should I inseminate cow x?) when they believe it will have positive economic impact.” This may highlight another reason why PLF technologies are more commercially established in the dairy sector: the benefits of using PLF are much clearer, whereas the advantages of using PLF in poultry farming have not yet been sufficiently demonstrated. Although PLF has the potential to improve bird health and welfare, its actual benefits, over and above those that could be obtained by simpler methods such as water use or greater attention to temperature and humidity, have not yet been demonstrated in practice.

More publications had animal health and welfare as one of the goals (63.64% of publications) than production (51.14%) (publications could have more than one goal). Likewise, for the publications with only one goal, more publications had animal health and welfare as the only goal (39.77%), compared to production (27.27%). This suggests that the majority of PLF development in poultry farming thus far has focussed on improving welfare. However, a substantial amount of research (almost a third) is focussing on production alone; in these cases, there could be a danger that production is prioritised over health and welfare. It should be noted that, where the goal of the study was not explicitly stated, it was inferred by the author (ER), which introduces a degree of subjectivity into the categorisation of study goal; this should be taken into account when interpreting these results.

In the absence (to the authors’ knowledge) of equivalent reviews, it is hard to compare the goals of PLF development in other livestock sectors. Taking the dairy sector again as an example, there are at least 11 commercially available accelerometers for oestrous detection, but only two commercially available sensors for lameness detection [50]; this could suggest that improving production processes has received more emphasis than welfare monitoring. However, there has been work focused on improving dairy cattle welfare through PLF technologies; for example, DairyCare was a 4-year (2014 to 2018) EU project with an objective of improving dairy animal well-being through technological advancement, including the development of biomarker-based, activity-based and systems-level welfare monitoring technologies [50].

Of the papers with animal health and welfare as the sole primary goal, most of the measurements used to monitor the birds were locomotory behaviour-based (43.81%). Locomotory behaviour included activity, distribution and occupation patterns (e.g., [48,53]), movement (e.g., [54]) and movement-related variables such as speed, step frequency, step length and the lateral body oscillation [55], location within the environment (e.g., [30]), optical flow (e.g., [56]), ranging behaviour (e.g., [57]), and clustering behaviour ([58]). The second largest proportion of publications (20.95%) used vocalisations [59] or bird sounds. Bird sounds were pecking sounds (e.g., [60]), or in one publication, rale sounds [61].

Other behaviour measures used were perching behaviour (e.g., [62]) and resting behaviour which included lying events [63,64] and latency to lie down [63]. Body posture was used to develop automated techniques of recognising and quantifying bird behaviours [65] such as wing spreading, scratching and preening [66]. The presence of birds was used by Zaninelli and colleagues [67,68] to detect whether hens in a free-range system were present in the housing area, with the aim of removing all hens to the outside area so the housing can be treated to reduce atmospheric ammonia and bacterial load. Monitoring of the presence of hens was also used to detect multiple occupations of a nest area to improve monitoring of laying behaviour [69]. Li and colleagues [70,71] used presence of hens at specific areas (feeding trough and nest boxes) for automated monitoring and quantification of feeding, drinking and nesting behaviour. Automated monitoring of hen presence was also used in an environmental preference test [72].

Welfare measures that focus on assessing the behaviour of animals are known as animal-based measures, or ’outcome’ measures [73]. These measure the animals directly and inform us of the effect (outcome) of an animal’s environment on its welfare state [74]. Outcome measures are considered to provide a more objective, accurate and direct picture of animal welfare than ’input’ measures, which describe what must be provided to animals in terms of housing, space, feed and water, veterinary care and management practices [74]. The use of outcome measures is considered best practice in welfare assessment schemes [73]. Furthermore, a ’continuous improvement’ approach is also considered best practice in welfare assessment schemes; this requires “regular monitoring of pre-defined criteria” ensuring that “preventive and corrective action is taken to maximise levels of these criteria” [73]. Therefore, most of the PLF systems proposed with the sole goal of improving welfare could help achieve best practice welfare assessment by continuously monitoring outcome measures in real-time, in order for preventive and corrective action to be taken.

However, to use behaviour as a welfare measure, the behaviours must be validated to show whether and how they are linked to an animal’s welfare status. For example, Fernandez and colleagues [48] compared data on locomotory behaviour to measures collected via a validated assessment protocol (Welfare Quality^®^). They found statistically significant correlations between locomotory behaviour (activity and occupation patterns) and welfare scores (for footpad lesions and hock burn), indicating that activity and occupation patterns are valid indicators of broiler welfare status.

This highlights a further potential impediment to PLF development. PLF technology can be used to monitor many parameters, such as behaviour, but whether and how the measurements taken by a PLF system are linked to a parameter, i.e., the internal validity of the measure (for example, how optical flow patterns are linked to broiler behaviour [56]), is not always clear. Furthermore, whether and how the monitored parameters are linked to welfare, i.e., the external validity of the measure (for example, what different patterns of optical flow tell us about the birds’ welfare status) must be established. Finally, the levels of this parameter at which interventions are necessary to improve welfare, and what interventions are appropriate, must also be determined. Therefore, while there is the potential for PLF to lead to improved standards of bird welfare through continuous, real-time monitoring, important steps before such systems can become successfully commercialised are internal and external validation by controlled trials to show that the system does in practice reliably monitor the parameter of interest, and that monitoring this parameter and appropriate intervening does lead to better welfare.

Most of the physiological measures made by PLF systems were body temperature of birds measured directly (e.g., [75]) as well as via thermal imaging (e.g., [76]). Environmental measures included temperature and humidity (e.g., [77]), CO_2_ concentration [78], moisture content of litter [79] and citric acid concentration in drinking solutions [80]; these can be considered as input measures of welfare.

The results of this review suggest that future work on PLF technologies for poultry farming should focus on overcoming barriers to commercialisation and on expanding the range of welfare measures, particularly those involving behaviour, that can be used as part of PLF. There is a need for more large-scale commercial trials that involve manufacturing companies, farmers and other stakeholders from the outset, in order to demonstrate the value of PLF systems in raising standards of welfare in practice. Ensuring that PLF technologies are transferred from the lab to the farm was the overall objective of the EU-PLF project, which ran from 2012 to 2016 [81]. The main output of the project was the EU-PLF Blueprint: a manual with pragmatic guidance on how to implement PLF systems at the farm level [82]. The publication of the EU-PLF Blueprint may increase the success of commercialising poultry PLF technologies. Future work could incorporate the use of the Blueprint; instructions on how to access and use the manual are provided in Guarino et al. [82].

In addition, as well as using PLF technology to assess animal welfare in order to prevent and alleviate poor welfare states, future research should focus on using PLF to promote positive welfare and provide food animals with “a good life”, or at least “a life worth living” [83]. For example, Daniel Berckmans and Thomas Norton, two pioneers of PLF, explain that “the possibilities to use PLF technology to create an interesting, adventurous environment for curious animals is not so much used yet, so there is another opportunity here to realise playful events and environments for our animals to give them a life that is worth living” [84].

## 5. Conclusions

The development of PLF systems for poultry farming, especially broilers and laying hens, has received increasing attention on a global scale, notably in the USA, China and Belgium, and the largest proportion of studies have focussed on improving animal health and welfare. Despite the increasing research output, PLF is yet to become a widespread commercial reality in the poultry sector. Future work should focus on the commercialisation of PLF systems in the poultry sector, as well as their potential for promoting positive welfare.

## Figures and Tables

**Table 1:** Search terms used in a systematic review of the use of precision livestock farming technologies in poultry farming.

Precision Livestock Farming Terms	Poultry Terms
Acoustic monitoring	Broiler
Audio signal processing	Chick
Automated monitoring	Chicken
Automated welfare	Duck
“Big data”	Goose
Biosensor	Hatchery
Control chart	Laying hen
“Image analysis”	Poultry
“Infrared thermal imaging”	Pullet
“Infrared thermal image”	Slaughter
“Infrared thermography”	Turkey
“Integrated management system”	
Intelligent farming	
Machine vision	
“Noise analysis”	
Optical flow	
PLF ^1^	
Precision agriculture	
“Precision feeding”	
Precision livestock farming	
Precision nutrition	
“Real-time monitoring”	
RFID ^2^	
Sensor	
“Signal analysis”	
Smart farming	
“Sound analysis”	
“Transmission color value”	
“Transmission colour value”	
UWB ^3^	
Vocali?ation analysis ^4^	
Wireless	

^1^ PLF = precision livestock farming. ^2^ RFID = radio frequency identification. ^3^ UWB = ultra-wideband. ^4^ A “?” was used to replace a single character to account for UK and US spelling variations, except in PubMed where such spelling variations are automatically included.

**Table 2:** Categories for extracting study characteristics from results of a systematic review of the use of precision livestock farming technologies in poultry farming.

Classification	Categories
Bird type	Broiler, Chicken ^a^, Duck, Goose, Laying hen, Poultry ^b^, Turkey
Country	Country of author affiliation, other (*n* < 5)
Income grouping of country	High-income, upper-middle-income, lower-middle-income, low-income
Goal	Animal health and welfare, Human health, Production, Sustainability
Prototype or commercially available system	Commercially available, Prototype
Technology	Camera, Control chart, Data management system, Incubator, Microphone, Precision feeding system, Robot, Sensor
Year	Year paper was published

^a^ The category chicken was only used when the paper did not specify broiler or laying hen; ^b^ The category poultry was only used when the available text did not specify the species further.

**Table 3:** Number and percentage of publications by country of author affiliation. Publications could have authors from more than one country.

Country	*n*	%
USA	50	18.94%
Belgium	49	18.56%
China	45	17.05%
Brazil	25	9.47%
UK	24	9.09%
Netherlands	21	7.95%
Italy	20	7.58%
Canada	12	4.55%
Iran	10	3.79%
Japan	10	3.79%
Turkey	9	3.41%
Germany	7	2.65%
Australia	6	2.27%
Spain	6	2.27%
France	5	1.89%
India	5	1.89%
Indonesia	5	1.89%
Other	36	13.64%

**Table 4:** Number and percentage of publications by country income grouping. Publications could have authors from more than one income grouping.

Country Income Grouping	*n*	%
High	232	87.88%
Upper-middle	100	37.88%
Lower-middle	13	4.92%
Low	0	0.%

**Table 5:** Number and percentage of publications by technology used. Publications could use more than one type of technology.

Technology	*n*	%
Sensor	137	51.89%
Camera	112	42.42%
Microphone	37	14.02%
Scales	12	4.55%
Robot	5	1.89%
Control chart	3	1.14%
Data management system	1	0.38%

**Table 6:** Number and percentage of publications by bird type. Publications could have more than one bird type.

Bird Type	*n*	%
Broiler	114	43.18%
Laying hen	67	25.38%
Chicken	41	15.53%
Poultry	31	11.74%
Duck	7	2.65%
Turkey	5	1.89%
Goose	1	0.38%

**Table 7:** Number and percentage of publications according to the goal(s) of the study. Publications could have more than one goal.

Goal	*n*	%
Animal health and welfare	168	63.64%
Production	135	51.14%
Sustainability	20	7.58%
Human health	10	3.79%
Unknown	3	1.14%

**Table 8:** Number and percentage of publications with animal health and welfare as the only goal (n = 105) according to the parameter(s) that were measured by the precision livestock farming system.

Parameter(s) Measured	*n*	%
Locomotory behaviour	46	43.81%
Vocalisation/bird sound	22	20.95%
Physiology	13	12.38%
Acceptability	9	8.57%
Presence of bird	6	5.71%
Environmental	6	5.71%
Perching behaviour	3	2.86%
Body posture	2	1.90%
Resting behaviour	2	1.90%

## Appendix A

**Table A1:** A full list of all countries of author affiliation, and number and percentage of publications with authors affiliated with these countries. Publications could have authors from more than one country.

Country	*n*	%
Austria	1	0.38%
Australia	6	2.27%
Bangladesh	1	0.38%
Belgium	49	18.56%
Brazil	25	9.47%
Bulgaria	1	0.38%
Canada	12	4.55%
China	45	17.05%
Denmark	3	1.14%
France	5	1.89%
Germany	7	2.65%
Greece	1	0.38%
Hungary	0	0.00%
India	5	1.89%
Indonesia	5	1.89%
Iran	10	3.79%
Ireland	2	0.76%
Israel	1	0.38%
Italy	20	7.58%
Japan	10	3.79%
Jordan	1	0.38%
Malaysia	4	1.52%
Netherlands	21	7.95%
Norway	1	0.38%
Pakistan	1	0.38%
Philippines	1	0.38%
Portugal	1	0.38%
Russia	2	0.76%
Saudi Arabia	1	0.38%
Slovakia	1	0.38%
South Africa	1	0.38%
South Korea	2	0.76%
Spain	6	2.27%
Sweden	3	1.14%
Switzerland	1	0.38%
Taiwan	4	1.52%
Thailand	2	0.76%
Turkey	9	3.41%
UK	24	9.09%
USA	50	18.94%
